# Gender differences in physical activity and sedentary behavior of Japanese primary school children during school cleaning time, morning recess and lunch recess

**DOI:** 10.1186/s12889-019-7256-5

**Published:** 2019-07-23

**Authors:** Chiaki Tanaka, Maki Tanaka, Shigeru Inoue, Masayuki Okuda, Shigeho Tanaka

**Affiliations:** 1 College of Health and Welfare, J. F. Oberlin University, 3758 Tokiwamachi, Machida, Tokyo, 194-0294 Japan; 2 Department of Early Childhood Education, Kyoto Bunkyo Junior College, 80 Senzoku, Makishima-cho, Uji, Kyoto, 611-0041 Japan; 3 Department of Preventive Medicine and Public Health, Tokyo Medical University, 6-1-1 Shinjuku, Shinjuku-ku, Tokyo, 160-8402 Japan; 4 Department of Environmental Medicine, Graduate School of Science and Engineering, Yamaguchi University, 1-1-1 Minamikogushi Ube, Yamaguchi, 755-8505 Japan; 5 Department of Nutrition and Metabolism, National Institute of Health and Nutrition, National Institutes of Biomedical Innovation, Health and Nutrition, 1-23-1 Toyama, Shinjuku-ku, Tokyo, 162-8636 Japan

**Keywords:** Active play, School life, Accelerometer, Sitting

## Abstract

**Background:**

The school environment provides crucial opportunities for children to engage in health-promoting physical activity (PA). Japanese children clean their schools and have recess time every school day. The primary aim of the study is to describe the levels of physical activity during school cleaning and recess time while comparing it between genders.

**Methods:**

PA and sedentary behavior (SB) of 230 boys and 252 girls aged 6–12 years-old from 14 public primary schools were assessed for 7 consecutive days with a triaxial accelerometer. Minutes of SB, and moderate-to-vigorous PA (MVPA) as a percentage in each of school cleaning time (15 min/day: 58% of the 14 schools or 20 min/day), morning recess time (15 min/day: 6% or 20 min/day) or lunch recess time (15 min/day: 29%, 20 min/day: 47% or 25 min/day) were evaluated. A one-way analysis of variance or Student’s t test was used to examine differences in %PAs and %SB between school cleaning time and morning and lunch recess time, and between genders.

**Results:**

In a school day, the percentage of total daily MVPA during school cleaning time, morning recess and lunch recess time was 19.4 ± 6.8% (15.2 ± 5.3 min/day) for boys and 16.9 ± 5.8% (10.5 ± 4.3 min/day) for girls. For boys, the proportions of MVPA in morning and lunch recesses and SB during the morning recess were significantly higher than during school cleaning time (*p* < 0.001). For girls, similar results were obtained and the SB during lunch recess was also higher than during school cleaning time (*p* < 0.001).

**Conclusions:**

These findings suggest that the total amount of school cleaning time, recess and lunch time likely contribute to daily MVPA but the beneficial effects should be further explored in future intervention studies.

**Electronic supplementary material:**

The online version of this article (10.1186/s12889-019-7256-5) contains supplementary material, which is available to authorized users.

## Background

Children and adolescents are recommended to participate in at least 60 min of moderate-to-vigorous physical activity (MVPA) daily to benefit health [1]. Japanese Children spend approximately 200 days/year in primary school [2]. According to results of a previous study of Japanese primary school children, time in MVPA during a typical school day was higher and time in sedentary behavior (SB) during a school day was lower than that during summer vacation [3]. The school environment plays a crucial role in providing opportunities for children to engage in health-promoting physical activity (PA) [4, 5]. Thus, previous reviews have focused on PA during school recess time [6–8].

Japanese educational curriculum guidelines for primary schools as established by the Ministry of Education, Culture, Sports, Science and Technology, Ministry of Health, Labour and Welfare require that school curricula include school cleaning time that is intended to increase understanding by primary school children of the significance of shared responsibility in school and work. Thus, Japanese children clean their schools (e.g., classroom, bathroom, hall, stairs, entrance, gym) themselves every school day [9]. Recently, Butte et al. developed A Youth Compendium of Physical Activities (Youth Compendium). Many cleaning activities in primary school children are applicable to activity as MVPA [10]. Thus, school cleaning time can be an opportunity for levels of PA, there is no evidence concerning PA during the school cleaning time for primary school children.

Hallal et al. described overall PA levels worldwide using questionnaire data for adolescents from 105 countries [11]. The data show that boys are more active than girls. Other international comparison studies also showed similar results [12]. According to a report from the Tokyo Metropolitan Board of Education, daily step counts for primary school children in the Tokyo metropolitan area [13] were lower for girls than those of boys in each grade (for the first grade, 10775 and 12733 steps/day, while for the sixth grade, 9134 and 12242 steps/day for girls and boys, respectively). Consequently, interventions and strategies that effectively increase children’s engagement in PA that benefits health outcomes are needed, especially for girls. Therefore, the primary aim of the study is to describe the PA levels during school cleaning and recess time. The secondary aim is to identify gender differences of them.

## Methods

One hundred and eighty-six boys and 223 girls participated from 14 primary schools in urban areas in Tokyo and Kyoto in this cross-sectional study. Participants were invited using a leaflet and all participants and their parents gave their written informed consent. The study protocol was approved by the Ethical Committee of J. F. Oberlin University (receipt number: 12023). Data were collected between June 2012 and January 2015 during the school year.

### Objective evaluation of sedentary behavior and physical activity

Participants wore a triaxial accelerometer (Active style Pro HJA-350IT, Omron Healthcare, Kyoto) on their waist to evaluate SB and PA. The details and calibration of the accelerometer are described elsewhere [14]. In brief, the accelerometer can distinguish ambulatory and non-ambulatory activities and predict intensity of both types of PA. The epoch length was set at 10 s. The metabolic equivalents (METs) provided by the accelerometer are overestimated for primary school children, as the prediction algorithms were derived for adults. Therefore, the following conversions were made, based on the results of Hikihara et al. [14]:

### Ambulatory activities

$$ {\displaystyle \begin{array}{c}0.6237\times \mathrm{MET}\ \mathrm{value}\ \mathrm{provided}\ \mathrm{by}\ \mathrm{the}\ \mathrm{Active}\ \mathrm{style}\ \mathrm{Pro}+0.2411\\ {}{\mathrm{R}}^2=0.772\end{array}} $$

### Non-ambulatory activities

$$ {\displaystyle \begin{array}{c}0.6145\times \mathrm{MET}\ \mathrm{value}\ \mathrm{provided}\ \mathrm{by}\ \mathrm{the}\ \mathrm{Active}\ \mathrm{style}\kern0.28em \mathrm{Pro}+0.5573\\ {}{\mathrm{R}}^2=0.880\end{array}} $$

SB and PA were continuously monitored for 7 days. Participants were requested to wear the accelerometers except during dressing, bathing or swimming. Non-wear time was defined as periods with over 1 h of consecutive zero counts. The accelerometer data were analyzed between 7:00 and 21:00 to exclude sleep time, because many participants wore the accelerometer during sleep and sleep and SB cannot be distinguished from the acceleration. We included days in which 600 min (10 h) or more of time wearing the device had accrued. Class teachers of study participants were asked to log time tables during measurement periods in a log sheet. We identified morning, lunch and cleaning time for the measurement period in every participant using timetables by class teachers. If non-wear time was found during school cleaning time or morning or lunch recesses, the data were excluded from the analysis. Our analysis included participants with data from at least 3 school days and at least 3 days in school cleaning time and morning and lunch recesses were included in the analysis. Participants and their parents were asked about absences from school in a log sheet and history of conditions affecting PA, such as respiratory disease, heart disease or injury by the questionnaire during measurement periods.

### School cleaning time and morning and lunch recess context

Japanese children clean their schools (e.g., classrooms, bathrooms, halls, stairs, entrances, gyms, etc.) every day. Meanwhile, morning and lunchtime recesses are not directly supervised by teachers and principals. Lunchtime recess does not include time when lunch is eaten. A head teacher or a deputy head teacher at each school was asked to complete a questionnaire concerning school cleaning time, and morning and lunch recess schedules. They also were asked about school policy concerning morning recess and lunch recess: 1: On days with good weather, as a rule, students play in the school playground during morning and lunch recesses; 2: Students can choose the play area in the school playground or gym during morning and lunch recesses; 3: Students can be anywhere in their school during morning and lunch recesses; or 4: Others.

### Anthropometric measurements

As an anthropometric measurement, body height and weight were measured. The details of evaluation of weight status are described elsewhere [3].

### Analyses

The SB and PA per each time was calculated in METs, wherein the average number of school day minutes spent in SB (METs ≤1.5), light PA (LPA) (1.6 ≤ METs ≤2.9), MVPA (3.0 ≤ METs) and vigorous PA (VPA) (6.0 ≤ METs) was calculated for each individual, and then average values and percentages were calculated. PA status for ambulatory activity or non-ambulatory activity in intensity-specific categories (LPA, MPA, VPA and MVPA) is indicated. The relative contributions of school cleaning time, morning recess time, and lunch recess time to daily weekday PA were calculated as a proportion using: ((time in the activity intensity/total time in the activity intensity during the school day) × 100), and averaged over valid days.

Student’s t test was used to examine differences in physical characteristics of participants between boys and girls in each grade. A one-way analysis of variance was used to examine differences in %PA and %SB between genders in school cleaning time, morning recess, and lunch recess adjusted for grade, school and relative weight. The relationship between the two variables (school cleaning time vs morning recess time, lunch recess time or total recess time, and morning recess time vs lunch recess time) was evaluated by partial correlation analysis controlled for grade, school and relative weight. Statistical analysis was performed with IBM SPSS statistics 23.0 for Windows (IBM Co., Tokyo, Japan). All statistical tests were regarded as significant for *p*-values < 0.05.

## Results

### Study participant characteristics

From the initial sample of 569 participants, data of 87 participants were excluded due to missing data (no accelerometer data by the above-mentioned criteria described below [*n* = 36], revocation of agreement [*n* = 8], history of conditions affecting PA, such as respiratory or heart disease [*n* = 27], no questionnaire data [*n* = 16]). As a result, the sample of the present study comprised data from 482 children. There was no significant difference in the age and the relative weight between the participants and children who dropped out.

The characteristics of participants are presented in Table 1. The average duration of accelerometer wear time was much greater than the minimum criteria specified (at least 3 weekdays and 10 h), with an average of 4.5 days and 13.3 h for boys, and 4.5 days and 13.5 h for girls. The school cleaning time, recess and lunch time durations were 15 (58% of 14 schools) or 20 min/day, 15 (6%) or 20 min/day, and 15 (24%), 20 (50%) or 25 min/day, respectively. The days with school cleaning time, morning recess, and lunch recess were 4.0, 4.1 or 4.1 days for boys and 4.1, 4.2 or 4.2 days for girls, respectively. Ten schools (71% of 14 schools) in the present study answered: “1: On days with good weather, as a rule, students play in the school playground during morning and lunch recesses” as a school policy. The remaining four schools (29% of 14 schools) answered “3: Students can be anywhere in their school during morning and lunch recesses”.

**Table 1:** Physical characteristics of study participants and physical activity in daily school day by gender

Variables	Boys (n=230)			Girls (n=252)			Mean difference	95%CI		P-value
	Average		SD	Average		SD	(Boys-Girls)			
Age (years)	9.4	±	1.6	9.3	±	1.6	0.0	-0.2	0.3	0.813
Height (cm)	132.5	±	9.9	132.3	±	11.1	0.1	-1.8	2.0	0.902
Weight (kg)	29.8	±	7.7	28.7	±	7.1	1.1	-0.2	2.4	0.115
Body mass index (kg/m^2^)	16.7	±	2.5	16.1	±	1.9	0.6	0.2	1.0	0.006
Relative body weight (%)	-1.3	±	13.4	-3.5	±	10.7	2.2	0.1	4.4	0.043
Weight status (overweight and obese: %)	7.4			2.8						
Moderate-to-vigorous physical activity in daily school day (min/day)	81	±	22	62	±	18	18.4	15	22	<0.001

*95%CI* 95% Confidence Interval

Time spent in MVPA during the school day is shown in Table 1. The proportions of SB, different activity intensity levels for ambulatory and non-ambulatory activity, and total activity during school cleaning time, morning recess and lunch recess are displayed in Table 2 and, Additional file 1: Tables S1, S2 and S3. For boys, the proportions of MPA, VPA, and MVPA (morning recess time: 30.1 ± 14.1%, *p* < 0.001 or lunch recess time: 29.6 ± 12.4% vs. school cleaning time: 19.3 ± 8.2%, both *p* < 0.001) in morning and lunch recesses and SB in morning recess time were significantly higher than that during school cleaning time (17.4 ± 11.7% vs. 13.8 ± 9.9%, *p* < 0.001), respectively. On the contrary, the duration of LPA during morning and lunch recesses was lower than that during school cleaning time. In girls, similar trends were observed. However, the proportions of boys’ MPA, VPA and MVPA in each time were higher than those of girls (*p* < 0.001). The results of an analysis of covariance adjusted for school, grade and relative weight indicated that proportions of MPA, VPA, and MVPA during school cleaning time and morning and lunch recesses were higher for boys than for girls (*p* < 0.001) and the proportions of boys’ SB in school cleaning time (*p* = 0.011) and morning and lunch recesses time (*p* < 0.001) were lower than those of girls (*p* < 0.001). LPA occupied the largest proportion of both school cleaning time and morning and lunch recesses for boys and girls. The proportions of boys’ LPA in morning and lunch time were lower than those of girls (*p* < 0.001).

**Table 2:** Sedentary behavior and physical activity during school cleaning time and morning and lunch recesses by gender

Variables	School cleaning time (%: each time)										Morning recess (%: each time)										Lunch recess time (%: each time)										Boys	Girls
	Boys			Girls			Mean difference	95%CI		P-value	Boys			Girls			Mean difference	95%CI		P-value	Boys			Girls			Mean difference	95%CI		P-value		
	Average		SD	Average		SD	(Boys-Girls)				Average		SD	Average		SD	(Boys-Girls)				Average		SD	Average		SD	(Boys-Girls)					
Sedentary behavior	13.8	±	9.9	16.2	±	11.7	-2.5	-4.4	-0.6	0.011	17.4	±	11.7	23.3	±	13.8	-6.0	-8.2	-3.7	<0.001	15.1	±	11.1	18.9	±	9.8	-3.9	-5.7	-2.0	<0.001	a, c	a, b, c
LPA																																
Ambulatory	36.0	±	9.7	30.3	±	9.9	5.8	4.0	7.5	<0.001	26.9	±	7.5	23.3	±	7.9	3.6	2.3	5.0	<0.001	29.5	±	7.8	25.9	±	7.5	3.6	2.2	4.9	<0.001	a, b, c	a, b, c
Non-ambulatory	27.8	±	8.7	34.4	±	9.6	-6.7	-8.3	-5.0	<0.001	23.5	±	9.4	31.3	±	9.7	-7.9	-9.6	-6.2	<0.001	23.5	±	9.0	32.8	±	8.7	-9.3	-10.9	-7.8	<0.001	a, b	a, b, c
Total time	63.8	±	8.4	64.6	±	9.0	-0.9	-2.5	0.7	0.262	50.4	±	9.1	54.6	±	9.8	-4.3	-5.9	-2.6	<0.001	53.0	±	9.0	58.8	±	8.9	-5.8	-7.2	-4.3	<0.001	a, b, c	a, b, c
MPA																																
Ambulatory	10.8	±	5.9	7.5	±	5.3	3.3	2.3	4.3	<0.001	19.0	±	9.9	11.2	±	7.1	7.9	6.4	9.4	<0.001	19.6	±	9.6	11.8	±	6.5	7.8	6.3	9.2	<0.001	a, b	a, b
Non-ambulatory	6.7	±	3.8	7.3	±	7.3	-0.5	-1.2	0.2	0.155	4.9	±	2.9	4.7	±	2.6	0.1	-0.3	0.6	0.604	5.1	±	2.9	5.5	±	3.0	-0.3	-0.9	0.2	0.181	a, b	a, b, c
Total time	17.5	±	7.1	14.8	±	7.3	2.8	1.6	4.1	<0.001	23.9	±	10.0	16.0	±	8.0	8.0	6.4	9.6	<0.001	24.7	±	9.3	17.3	±	7.3	7.4	5.9	8.9	<0.001	a, b	a, b, c
VPA																																
Ambulatory	1.4	±	1.5	0.9	±	1.3	0.6	0.3	0.8	<0.001	4.8	±	4.7	2.9	±	3.7	2.0	1.2	2.7	<0.001	4.0	±	3.8	2.0	±	1.9	2.0	1.5	2.6	<0.001	a, b, c	a, b, c
Non-ambulatory	0.4	±	0.7	0.2	±	0.6	0.2	0.1	0.3	0.002	1.4	±	2.0	0.9	±	1.4	0.5	0.2	0.8	<0.001	0.9	±	1.1	0.5	±	0.8	0.4	0.2	0.6	<0.001	a, b, c	a, b, c
Total time	1.9	±	2.0	1.1	±	1.9	0.7	0.4	1.1	<0.001	6.2	±	6.2	3.8	±	4.8	2.5	1.5	3.5	<0.001	4.9	±	4.4	2.5	±	2.5	2.4	1.8	3.1	<0.001	a, b, c	a, b, c
MVPA																																
Ambulatory	12.2	±	6.8	8.4	±	6.2	3.9	2.7	5.1	<0.001	23.8	±	13.1	14.0	±	9.4	9.9	7.9	11.9	<0.001	23.5	±	12.3	13.8	±	8.0	9.8	8.0	11.6	<0.001	a, b	a, b
Non-ambulatory	7.1	±	4.0	7.5	±	4.3	-0.3	-1.0	0.4	0.385	6.3	±	3.5	5.6	±	3.2	0.6	0.1	1.2	0.028	6.1	±	3.1	6.0	±	3.2	0.0	-0.5	0.6	0.886	a, b	a, b
Total time	19.3	±	8.2	15.9	±	8.4	3.6	2.1	5.0	<0.001	30.1	±	14.1	19.7	±	11.0	10.5	8.3	12.7	<0.001	29.6	±	12.4	19.8	±	9.0	9.8	7.9	11.8	<0.001	a, b	a, b

*LPA* light physical activity, *MVPA* moderate-to-vigorous physical activity, *VPA* vigorous physical activity, *MPA* moderate physical activity, mean difference, *95%CI* 95% Confidence Interval and P-value were adjusted grade, school and relative weight, different alphabets show significant differences; a: School cleaning time VS Morning recess, b: School cleaning time VS Lunch recess time, c: Morning recess VS Lunch recess time, P<0.05

Table 3 shows the partial correlations between percentages of MVPA in school cleaning time and morning or lunch recess, controlling for age, relative weight, and wear time. There were significant, but very weak correlations between these factors for boys (*r* = 0.14 to 0.19). On the other hand, there were weak or moderate correlations for girls (cleaning time VS, morning recess time: *r* = 0.28, lunch recess time: *r* = 0.43, or total recesses time: *r* = 0.40).

**Table 3:** Partial correlation coefficients between moderate-to-vigorous physical activity during school cleaning time and during recesses

Variables	School cleaning time (%: each time)	P-value	Morning recess time (%: each time)	P-value
Boys				
Morning recess time (%: each time)	0.14	0.034	-	
Lunch recess time (%: each time)	0.18	0.007	0.43	<0.001
Total recesses time (%: each time)	0.19	0.004	-	
Girls				
Morning recess time (%: each time)	0.28	<0.001	-	
Lunch recess time (%: each time)	0.43	<0.001	0.47	<0.001
Total recesses time (%: each time)	0.40	<0.001	-	

adjusted for grade, school and relative weight

**Fig. 1:**
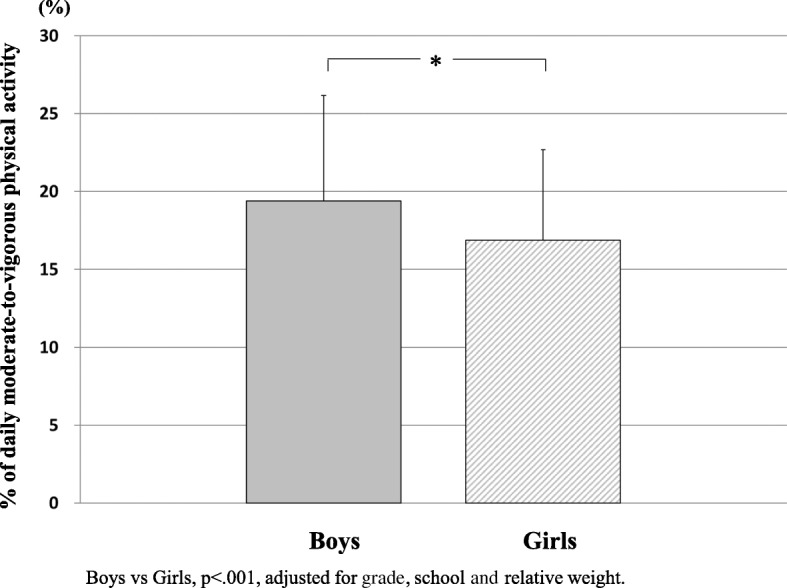
Contribution of moderate-to-vigorous physical activity during school cleaning time, recess and lunch time. Boys vs girls, *p* < 0.001

Figure 1 shows the contribution of MVPA during school cleaning time, recess and lunch time to total MVPA time of the school day by gender. Percentages of total MVPA in school cleaning time, recess and lunch time to total MVPA in the school day were 19.4 ± 6.8% (15 ± 5 min/day) for boys and 16.9 ± 5.8% (10 ± 4 min/day) for girls.

## Discussion

This study described the duration of SB and PAs classified as ambulatory and non-ambulatory activity measured using triaxial accelerometry during school cleaning time, and morning and lunch recesses in Japanese primary school children. Gender differences were also identified. For boys, the proportions of MPA, VPA, and MVPA in morning and lunch recesses and SB in morning recess time were significantly higher than those during school cleaning time and the LPA during morning and lunch recesses was lower than those during school cleaning time. For girls, similar results were obtained and SB during lunch time was higher than those during school cleaning time. Moreover, the percentage of total MVPA during school cleaning time and morning and lunch recesses in total MVPA on school days was 19.4 ± 6.8% per day on school days (15 ± 5 min/day) for boys and 16.9 ± 5.8% (10 ± 4 min/day) for girls. MVPA time during each of school cleaning time and morning and lunch recesses was not long but the total amount cannot be ignored.

Many studies have examined PA levels of young people during school recess times [7, 8]. However, to our knowledge, this is the first study in which the PA and SB during school cleaning time were examined. In a previous study of primary school children, using the respiratory measurements to examine METs for primary school children participating in the main activities in school cleaning time at Japanese schools (e.g., sweeping up, clearing away items and washing the floor), studies have found that the average MET values for these three activities were 3.15 ± 0.73, 3.01 ± 0.58 and 4.41 ± 0.69 MET, respectively [14]. Recently, Butte et al. developed “A Youth Compendium of Physical Activities” to estimate the energy costs of PAs using data on youth only [10]. They showed many types of housekeeping were more than 3METs in primary school aged children (e.g. dusting, dusting and sweeping, hanging out washing, sweeping). Although school cleaning time can be an opportunity for MVPA, our research found no evidence concerning substantial PA during school cleaning time for primary school children under free-living conditions. The average percentage of total MVPA during school cleaning time in the present study was 19.3 ± 8.2% for boys and 15.9 ± 8.4% for girls (Table 2), which corresponds to about 2 to 3 min/day. Thus, these findings suggest that the contribution to MVPA was not high for either gender. If school cleaning time is 20 min per day and all the time is regarded as MVPA, total MVPA time may be overestimated by almost 15 min per day. During school cleaning time, the percentages of non-ambulatory MVPA were significantly higher than those in morning recess for girls, whereas total MVPA, ambulatory MVPA, and SB during school cleaning time were significantly lower than those in morning recess for both boys and girls and SB in lunch recess for girls. Thus, the contents of PA and SB differed between the school cleaning time and morning and lunch recesses. Surprisingly, for girls in the present study, the partial correlations of MVPA between school cleaning time and morning or lunch recesses were weak or moderate. On the other hand, there was little correlation for boys (Table 3). This result suggests that boys might distinguish their activities in terms of educational vs play.

In the present study, the percentage of time spent in MVPA during morning recess (20 to 30%) was similar to previous studies of 8–11 year-olds from Canada [15], 5–10 year-olds in the UK [16], 9–12 year-olds in Hungary [17], and third-sixth grade children in the United States [18]. However, another study reported MVPA of 63–78% based on evaluations of pedometer engagement in third-fifth grade children in the United States [19]. On the other hand, in the present study SB during morning recess (17% in boys and 23% in girls) was lower than that seen in the above-mentioned studies (from 35 to 64%) [15–17]. The percentage of time spent in SB during morning and lunch recess in the present study was similar to previous studies of 9–11 year-olds from the US (22%) during the recess/lunch time period [20]. Differences in methods used to assess PA may account, in part, for differences in our results relative to previous studies. In addition, except for four schools, all schools in the present study had the policy: “On days with good weather, as a rule, students play in the school playground during morning and lunch recesses” This policy might explain why the SB during morning recess was lower than that of previous studies.

A previous systematic review found that during school recess periods (recess and lunchtime), boys were consistently more active than girls [7]. The present study is consistent with these earlier results in that PAs for boys during lunch recess were higher than that for girls. However, there were no gender differences in non-ambulatory MPA among morning and lunch recesses and non-ambulatory MVPA among lunch recess times. The non-ambulatory LPAs and total LPA times for girls were higher than those for boys. Evidence from our study supports the assertion that both ambulatory activity and non-ambulatory activity are important factors in evaluating PA lifestyles in primary school children. These data may be particularly important for providing insight into gender as a determinant of PA in primary school children. Moreover, SB times for girls in the present study were longer than those for boys during morning and lunch recesses. Other previous studies reported that boys view recess as an opportunity to play competitive games that often dominate the available space [21]. In comparison, girls may view recess as an opportunity to socialize with friends [22]. The similarity in MVPA among boys and girls during morning recess, but lower amounts of SB for girls during lunch recess, indicates the need for future research to compare in greater detail the specific activities and behaviors of boys and girls during recesses. Identifying activities that differ by gender could be critical for the development of activity promotion strategies, and may help to inform evidence-based practices and policies designed to increase PA and decrease SB in primary school children.

The contribution of school cleaning time and morning and lunch recesses to daily MVPA for boys and girls was 19.4% (15 min/day) and 16.9% (10 min/day), respectively. These data suggest that cleaning time and morning and lunch recesses do not contribute significantly to overall daily PA levels on school days for primary school children. One reason for the low PA during morning or lunch recess was that the recess periods are only 15–20 min/day. A previous review reported that lunch time lengths in previous studies was 30–86 min/day (some studies that were considered included the time taken to eat lunch in the overall lunch period length), although the length of the morning recess in the present study (15–20 min/day) is similar to previous studies concerning schoolchildren in the UK, Belgium, Cyprus and the United States [23]. Parrish et al. [24] reported that the proportion of active children at each school and the actual number of minutes the children spent in the playground during the observational data collection period showed a positive correlation of *r* = .318 (*P* = .289). Moreover, the Tokyo Metropolitan Board of Education examined daily step counts for boys and girls who lived in Tokyo and found that step counts after school were higher than those during school [13]. Wood and Hall compared PA during morning playtime (15 min) and lunch playtime (60 min), and physical education classes, and found that time in MVPA was significantly longer during physical education than playtime (*P* < 0.01) [25]. Thus, future research should examine PA and SB of Japanese primary school children during the time after school and physical education classes.

There are several methodological points and limitations to be considered in the interpretation of these results. First, although the accelerometer Active style Pro is a widely used to measure PA and SB in Japan, it cannot provide information about the types of activities being performed or the context. Therefore, direct observational approaches for contextual PA and BS information are necessary. Second, our sample was not a representative for Japanese children. The strengths of our study include: an objective measure of SB and PA to classify ambulatory and non-ambulatory activity, and the use of a sample of Japanese primary school children from 14 schools. Notably, the present data were recorded in 10-s epochs, which may be sufficiently sensitive to detect short bursts of vigorous activity [26, 27]. Short time periods for data recording could be better reflect movement patterns of children.

## Conclusions

In summary, boys and girls accumulated 15 and 10 min of MVPA, respectively, during school cleaning time and morning and lunch recesses. Activity levels were higher during recess than during school cleaning time. Importantly, gender was found to be a moderator of PA behavior in school cleaning time and morning and lunch recesses, with boys exhibiting more VPA and MVPA than girls, although there were no gender differences in non-ambulatory MPA. These findings suggest that the total amount of school cleaning time, recess and lunch time likely contributes to daily MVPA but the beneficial effects should be further explored in future intervention studies.

## Additional file

Additional file 1: **Table S1.** Percentages of sedentary behavior and physical activity during school cleaning time by age group and gender. **Table S2.** Percentages of sedentary behavior and physical activity during morning recess time by age group and gender. **Table S3.** Percentages of sedentary behavior and physical activity during lunch recess time by age group and gender. (ZIP 104 kb)

## Data Availability

The data for this study are not publicly available due to lack of informed consent for data sharing at the time of collection, but are available from the corresponding author on reasonable request. For further information on the data and materials used in this study, please contact the corresponding author.

## Abbreviations

LPA: Light intensity activity
METs: Metabolic equivalents
MPA: Moderate physical activity
MVPA: Moderate-to-vigorous physical activity
PA: physical activity
SB: Sedentary behavior
VPA: Vigorous physical activity
